# Trends in the Aggressiveness of End-of-Life Cancer Care in the State of Qatar

**DOI:** 10.1200/JGO.2015.000620

**Published:** 2016-01-20

**Authors:** Azza A. Hassan, Hassan Mohsen, Ayman A. Allam, Pascale Haddad

**Affiliations:** **Azza A. Hassan**, **Hassan Mohsen**, and **Pascale Haddad**, Weill Cornell Medical College; **Azza A. Hassan** and **Ayman A. Allan**, National Center for Cancer Care and Research, Doha, Qatar; and **Azza A. Hassan**, Alexandria University, Egypt.

## Abstract

**Purpose:**

Quality of end-of-life (EOL) care is a key component of excellence in cancer care, and monitoring indicators for quality of EOL cancer care is crucial to providing excellent care. The aim of the current study is to describe the relative aggressiveness of EOL cancer care in the state of Qatar and to compare it with international figures.

**Methods:**

We analyzed all deaths from cancer in Qatar between January 1, 2009 and December 31, 2013. A total of 784 eligible patients were studied to assess aggressiveness of cancer care at EOL.

**Results:**

The average number of intensive care unit admissions per person decreased from 0.44 to 0.22 (*P* < .001) over the period of study. In addition, patients spent fewer days in the intensive care unit (2.79 to 1.82 days; *P* = .006) and made fewer visits to the emergency department (1.00 to 0.52 visits; *P* < .001) in the last 30 days of life. Fewer patients had at least one aggressive treatment measure at EOL during the 5-year period (82.3% to 71.0%; *P* = .038). The mean composite score for aggressiveness of EOL care decreased from 2.24 to 1.92 (*P* < .01).

**Conclusion:**

The aggressiveness of EOL cancer care has significantly decreased over time in Qatar; however, despite this decrease, the rate is still higher than that reported internationally. The integration of community palliative care services in Qatar may further decrease the aggressiveness of cancer care at EOL.

## INTRODUCTION

Despite advances in early detection, treatment, and survival, cancer remains a leading cause of death in most developed countries. With the number of deaths from cancer and other chronic diseases expected to increase as a result of a growing and aging population, the need for quality end-of-life (EOL) care has become increasingly important.^1^

During the past decade, cancer care at EOL has become increasingly aggressive,^2,3^ resulting in disproportionately greater health expenditures for decedents than for survivors.^2-5^ This increased treatment intensity, however, does not correspond to improved health outcomes and patient or family satisfaction,^6,7^ and raises concerns about the appropriateness of providing aggressive care before patients die. Furthermore, the emotional burden, loss of hope after experiencing the failure of life-sustaining treatments, and financial costs can exact a substantial toll from patients, families, and society.^8,9^

To systematically examine the quality of cancer care at EOL, Earle et al^10^ developed major indicators of poor-quality care. These indicators are a combination of measures of overly aggressive care and underuse of supportive care services, such as palliative care or hospice services. Indicators are defined as the last dose of chemotherapy received within 14 days of death, a new chemotherapy regimen starting less than 30 days before death, more than one emergency department (ED) visit within 30 days of death, more than one hospital admission or spending more than 14 days in hospital in the last month of life, an intensive care unit (ICU) admission in the last month of life, or death in an acute care hospital.

These indicators were adopted by researchers from several countries worldwide to illustrate the quality of care received in the last months of life by patients with cancer.^11-15^; however, such patterns of cancer care have never been explored in the Middle East. The significance of evaluating such trends in the state of Qatar is highlighted by three facts: cancer accounted for 18% of all deaths in Qatar from 2000 to 2012^15^; improving the quality of EOL cancer care is now recognized as a key factor for excellence in cancer care as a result of the Qatar universal health care system; and trends in the quality of cancer care at EOL can be analyzed in a population that encompasses all age and cancer groups, which facilitates comparison with international trends. These findings can be directly applied to inform national public health policies.

The purpose of this study, therefore, was to evaluate the aggressiveness of EOL cancer care in Qatar. A secondary end point was to compare our results with those observed worldwide.

## METHODS

### Data Sources

All deaths between 2009 and 2013 for which cancer was the underlying cause were extracted from the National Registry of Deaths Database. By law in Qatar, a death must be registered and the underlying cause of death recorded to approve the deceased for burial. The underlying cause of death was recorded in the database using the International Classification of Diseases, Tenth Revision, codes.

### Study Design and Study Population—Cohort Selection

This retrospective, population-based cohort study analyzed all adult cancer deaths in Qatar registered by the National Registry of Deaths Database from January 1, 2009 to December 31, 2013.

A retrospective chart review was conducted to measure for markers of aggressive EOL cancer care, defined as the occurrence of any of the following indicators: the last dose of chemotherapy received within 14 days of death, a new chemotherapy regimen started less than 30 days before death, more than one ED visit within 30 days of death, more than one hospital admission or spending more than 14 days in the hospital in the last month of life, an ICU admission in the last month of life, or death in an acute care hospital.

### Explanatory Variables

Explanatory variables included the following: age at death (reported as a continuous variable), gender (male *v* female), nationality (Qatari *v* other nationality), primary tumor site (lung, breast, colorectal, lymphoma, brain, prostate, or other), and place of death (palliative care, oncology unit, Hamad General Hospital; medical ICU; accident/emergency; brought-in-dead unit; and other).

### Main Outcome Measure

We developed indicators of aggressive care by identifying cutoffs rounded to clinically meaningful numbers. The following indicators were measured as described elsewhere^10^: started a new chemotherapy regimen in the last month of life, received the last dose of chemotherapy within 14 days of death, had more than one ED visit within 30 days of death, spent more than 14 days in the hospital at last encounter, had more than one hospitalization within 30 days of death, or had at least one ICU admission within 30 days of death.

We then created the main outcome measure, a categorical composite measure of aggressive EOL care, which was defined as the occurrence of at least one of the indicators of aggressive care. The categorical composite measure of aggressive EOL care was reported as the proportion of aggressiveness of care.

### Statistical Analyses

Descriptive statistics were summed as means and standard deviation of age and calculated as percentages for gender, nationality, primary site of cancer, and place of death. We then conducted an analysis of variance to test for differences in the mean age by year as well as a χ^2^ test to test for differences in proportions of the categorical variables by year. The Cochrane-Armitage test was used to determine significance of trends over time.

The Cochran-Armitage test was used to evaluate time trends, from 2009 to 2013, of the categorical composite measure of aggressiveness. A continuous composite measure of aggressive EOL care was reported as the average number of aggressive care measures by creating a score of those measures with a minimum score of not having any measure of aggressiveness and a maximum score of having all five measures of aggressiveness. Spearman rank-order correlation was used to evaluate time trends of the continuous composite measure of aggressive EOL. Results are reported in Table 1.

**Table 1:**
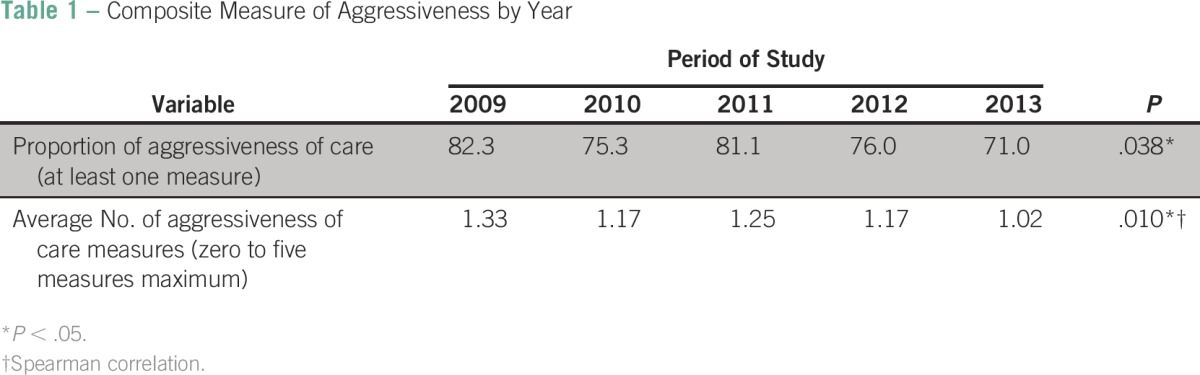
Composite Measure of Aggressiveness by Year

Multivariable logistic regression was used to examine the simultaneous effects of year of death, age, gender, nationality, primary tumor site, and place of death on the categorical composite measure of aggressive EOL care, that is, patients who experienced at least one indicator of aggressive care. Unadjusted odds ratios (UORs) and adjusted odds ratios (ORs) and corresponding 95% CIs were computed and are reported in Table 2.

**Table 2:**
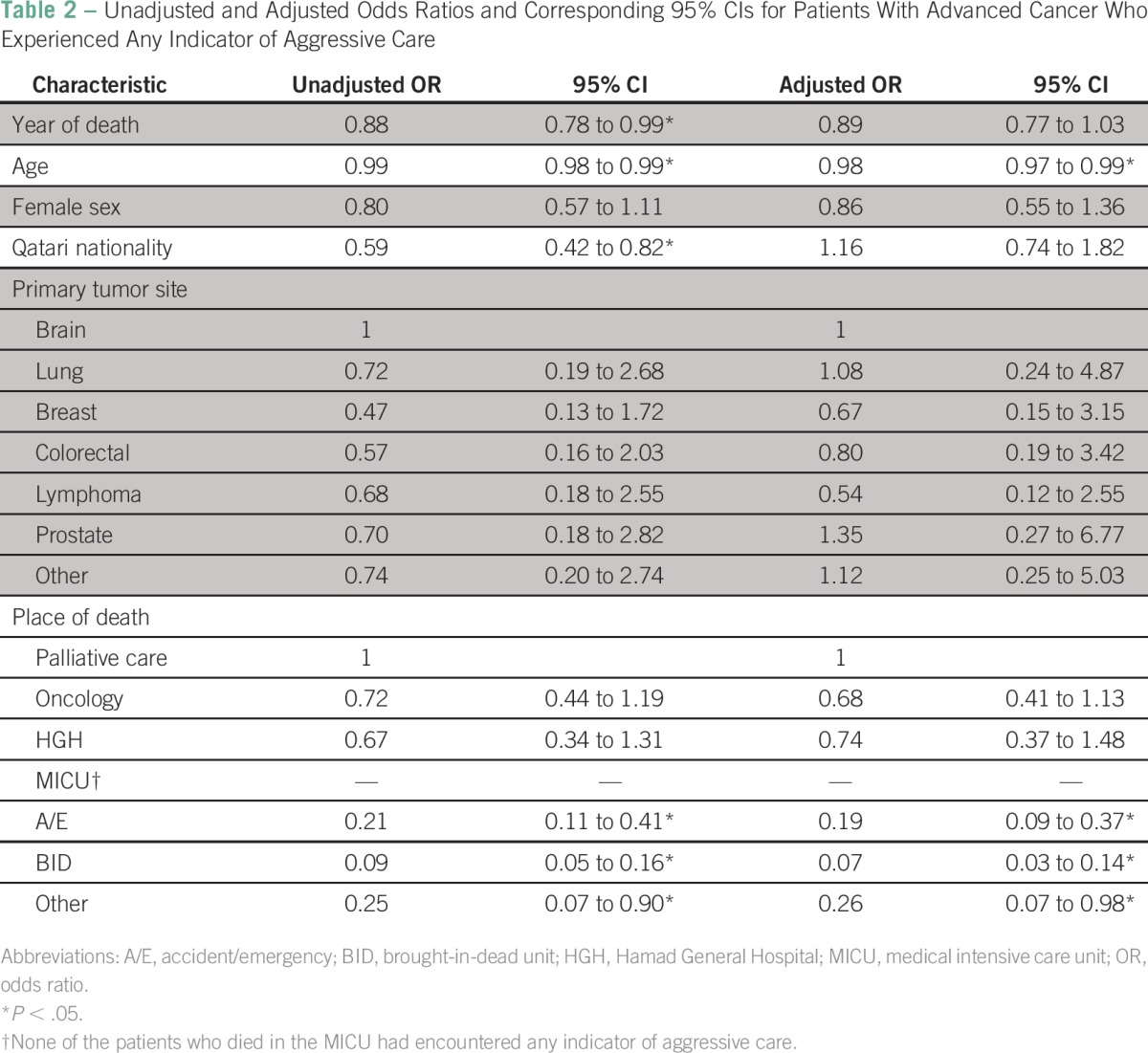
Unadjusted and Adjusted Odds Ratios and Corresponding 95% CIs for Patients With Advanced Cancer Who Experienced Any Indicator of Aggressive Care

All statistical analyses were conducted using STATA software version 13 (STATA, College Station, TX; Computing Resource Center, Santa Monica, CA). *P* < .05 was considered significant.

### Ethics and Permission

This study was approved by the Research Ethics Committee, Hamad Medical Corporation, Medical Research Center, Qatar.

## RESULTS

### Characteristics of Patients With Cancer by Year of Death

Table 3 shows the characteristics of expired patients with cancer during the 5-year study period of 2009 to 2013 who met the eligibility criteria, that is, patients who spent ≤ 31 days in the hospital upon last admission (N = 784). Two hundred forty-one patients (23.5%) spent more than 31 days in the hospital at last encounter and, thus, have been excluded from the analysis. The mean age during the study period was consistent, ranging from 57.2 years to 60.3 years (*P* = .221). During the 5-year period, there was no statistically significant difference in the proportion of females; approximately one-half of patients were female (range, 40.8% to 50.0%; *P* = .478). A smaller number of patients with a Qatari nationality was found in our sample throughout the years (range, 38.8% to 46.2%; *P* = .761). Colorectal was the most prevalent site of cancer, followed by lymphoma, lung, or breast cancer. There was no statistical significance by the year of study (*P* = .057). More patients died in the palliative care unit from 2011 on compared with earlier years, and a decreasing trend of death in the medical ICU by year of death was observed (*P* = .001).

**Table 3:**
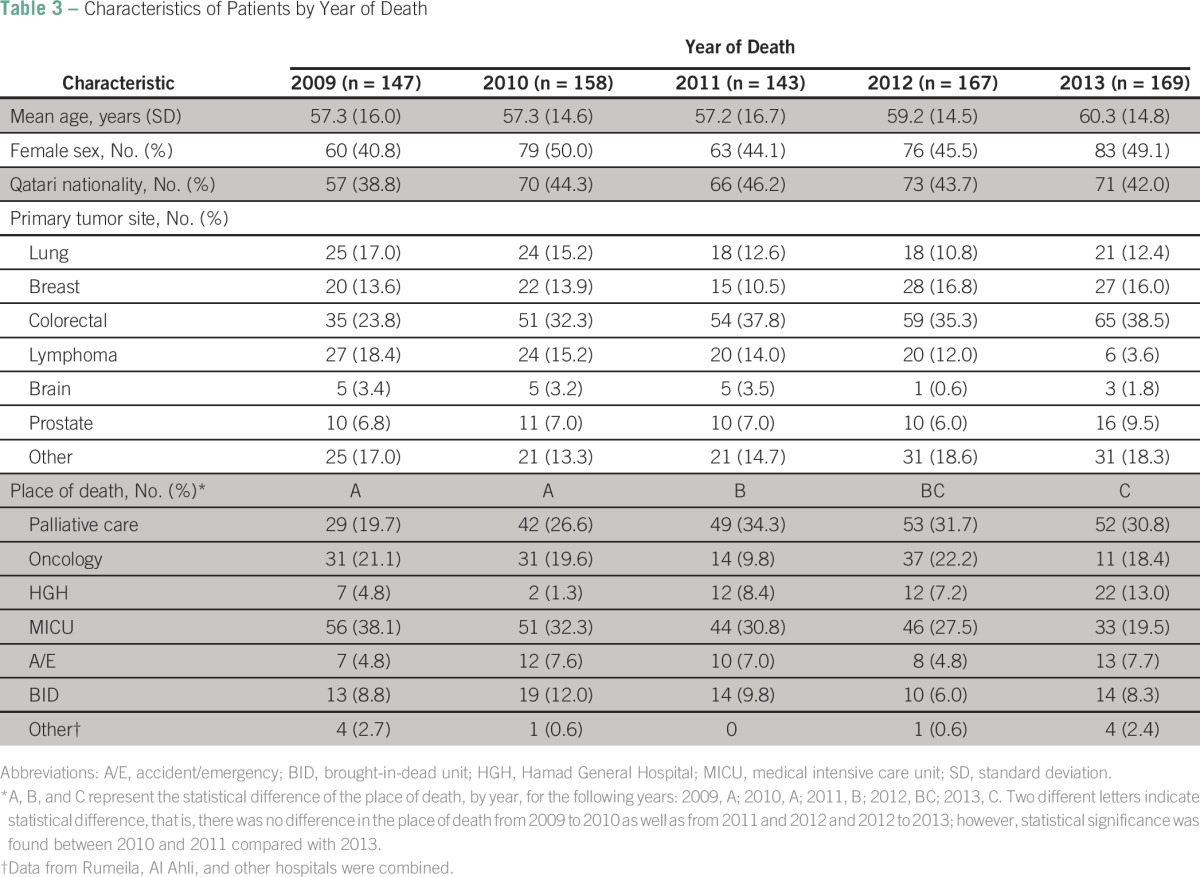
Characteristics of Patients by Year of Death

### Trends in Measures of Care and Indicators of Aggressive Care Among Patients With Cancer During the 5-Year Study Period

**Fig 1:**
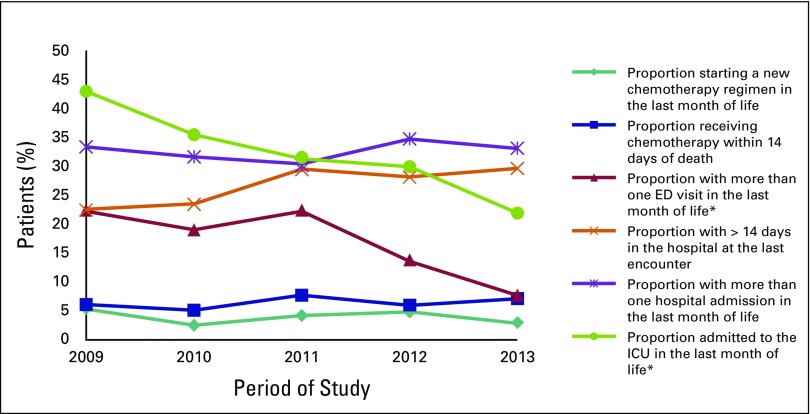
Trends for the six main indicators of aggressive end-of-life cancer care in Qatar during the 5-year study period, 2009-2013. ED, emergency department; ICU, intensive care unit. *P < .05.

Of the 784 patients with cancer included in the study period, the average number of ICU admissions between 2009 and 2013 decreased from 0.44 to 0.22 (*P* < .001; Fig 1 and Table 4). The same decrease was observed for the number of days spent in ICU in the last month of life in 2013 compared with 2009; the number of days in ICU decreased from 2.79 in 2009 to 1.82 in 2013 (*P* = .006; Fig 1 and Table 4). Moreover, there was, on average, one ED visit in the last month of life in 2009 compared with less than one ED visit in the last month of life in 2013 (*P* < .001; Fig 1 and Table 4). From 2009 to 2013, fewer patients had more than one ED admission in the last month of life (22.2% *v* 7.7%, respectively; *P* < .001; Table 5) or were admitted to the ICU (42.9% *v* 21.9%, respectively; *P* < .001; Table 5). However, between 2009 and 2013, the average number of days spent in the hospital at last encounter increased from 7.72 to 9.64 days (*P* = .029; Fig 1 and Table 4).

**Table 4:**
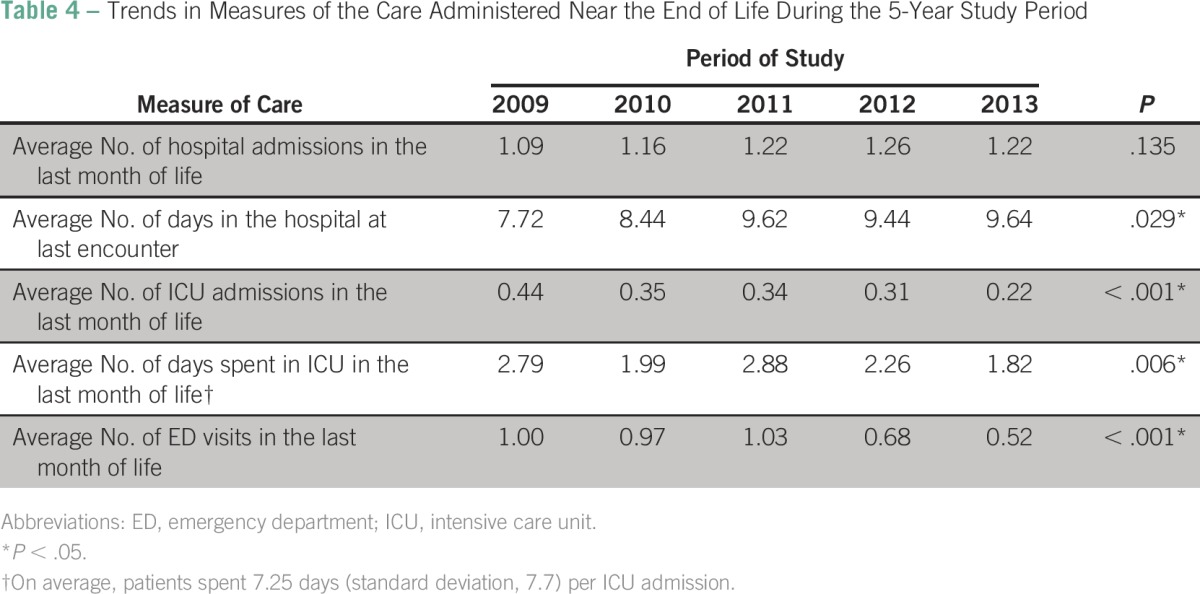
Trends in Measures of the Care Administered Near the End of Life During the 5-Year Study Period

**Table 5:**
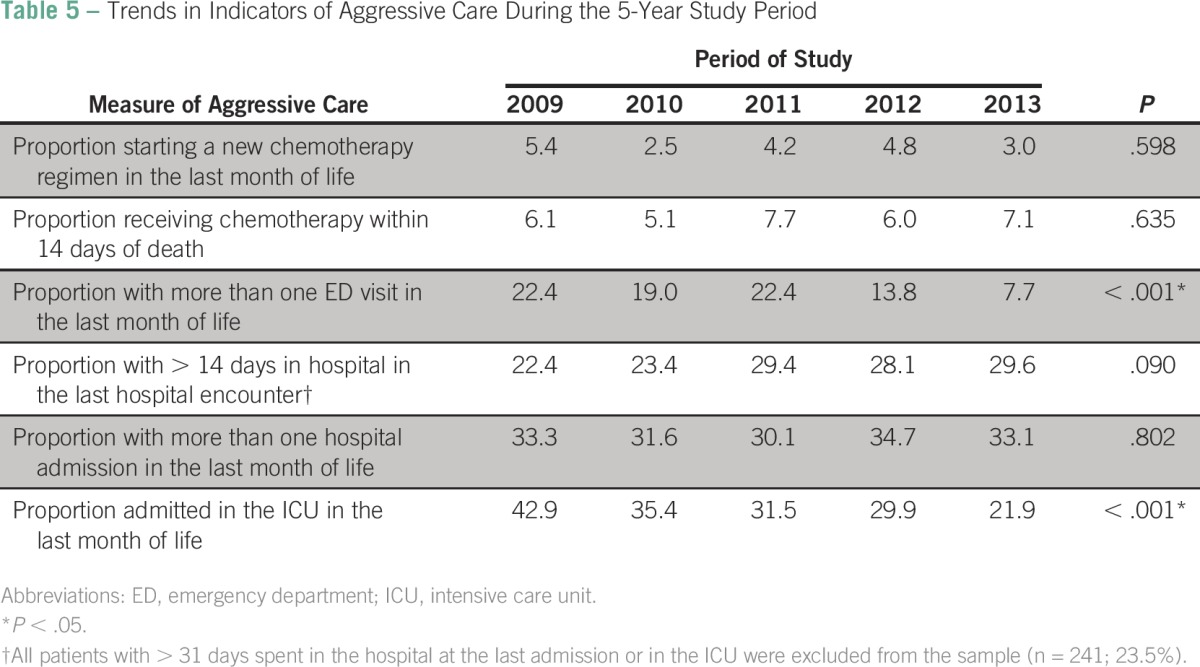
Trends in Indicators of Aggressive Care During the 5-Year Study Period

### Composite Measure of Aggressiveness by Year

Patients with at least one measure of aggressive care were considered to have been exposed to aggressiveness of care. The proportion of aggressiveness of care significantly decreased during the 5-year study period from 82.3% of patients exposed to at least one measure of aggressiveness to 71.0% (*P* = .038; Table 1). In addition, the average number of aggressive care measures, varying from zero measures per patient to five measures per patient, significantly decreased from 1.33 measures in 2009 to 1.02 in 2013 (*P* = .010; Table 1).

### Multivariable Logistic Regression

Table 2 shows UORs and ORs and corresponding 95% CIs for patients with cancer who experienced at least one measure of aggressive care in the last month before death. At the bivariable level, year of death was found to be a significant protective factor against aggressive care (UOR, 0.88; 95% CI, 0.78 to 0.99) as well as age of the patient (UOR, 0.99; 95% CI, 0.98 to 0.99) and being of Qatari nationality (UOR, 0.59; 95% CI, 0.42 to 0.82). No associations were found between gender or the primary site of cancer and aggressiveness of care. However, patients who died in the ED and the brought-in-dead unit were less likely to experience aggressive care (UOR, 0.21; 95% CI, 0.11 to 0.41; and UOR, 0.09; 95% CI, 0.05 to 0.16, respectively) compared with patients who died in the palliative care unit.

Multivariable logistic regression shows that older patients compared with younger patients (OR, 0.98; 95% CI, 0.97 to 0.99) and patients who died in the ED and the brought-in-dead units compared with patients who died in the palliative care unit (OR, 0.18; 95% CI, 0.09 to 0.36; and OR, 0.07; 95% CI, 0.03 to 0.14, respectively) were less likely to experience aggressive interventions.

## DISCUSSION

The current study showed a significant declining trend in the proportion of patients experiencing aggressive care at EOL, from 82% in 2009 to 71% in 2013 (*P* = .038). Despite this decline in the incidence of aggressiveness of care, 71% remains higher than percentages reported in the United States, Canada, Europe, and Asia.^5,7,9,16^

This is most likely attributable to many factors, including a lack of chronic care facilities (eg, hospice or home health care) to which patients can be referred when no acute symptoms require hospitalization as well as the lack of appropriate prognostication models that can accurately predict whether a patient will die within days, weeks, or months. In addition, delayed referral to palliative care and the inherent unease among oncology and hematology physicians over referring patients to palliative care deprives patients of a quality of life that focuses on important concerns at an advanced stage of disease, such as pain control, psychosocial support, and relief of spiritual distress.

The fact that the percentage of hematology patients included in our study is as high, relatively, as that reported in other studies (13%) could be a contributing factor to the higher percentage of aggressive care observed in our study. Hui et al^17^ reported on quality of EOL care for 113 patients with hematologic malignancies who were treated between 2009 and 2010 at MD Anderson Cancer Center. The sample from Hui et al^17^ represented 14% of the total population of patients with advanced cancer treated in the same center at the same period of time. Results from this retrospective cohort study showed that patients with hematologic malignancies received more aggressive care, fewer palliative care unit admissions, more ICU admissions, more ED visits, and more chemotherapy at EOL. The composite score for aggressiveness was significantly higher than that for solid tumors. As we have observed, hematology patients are usually treated aggressively until EOL.

It has been clearly demonstrated in publications from our center^18,19^ that, in Qatar, we have a low percentage of patients who die at home (0.4%). This figure is opposite to the percentages reported in Europe and North America where most of the patients with advanced cancer die at home, and emphasizes the need for a chronic care facility in our palliative care program. It also means that we have a high percentage of patients with end stage advanced disease who die in an acute hospital setting and, thus, receive greater exposure to possible aggressive measures near EOL.

Hong et al^11^ reported in 2013 on a cohort of patients with advanced gastric cancer who were treated with palliative chemotherapy in the last month of life. Hong et al^11^ showed that those patients had a significantly greater number of ICU admissions compared with patients with same diagnosis who did not receive any chemotherapy. In the same study, patients who received chemotherapy in the last month of life had a lower overall survival compared with those who did not receive chemotherapy. This finding emphasizes the fact that aggressive care is not usually reflected in an improved survival.

In the current study, we used internationally validated indicators (Table 5) to measure aggressive care at EOL; such indicators are widely used in the literature for the same purpose.^1,2,10,20,21^ To our knowledge, the current study is the first in the Middle East and the Gulf region that used a combination of six indicators to measure aggressiveness of care for patients with advanced cancer at the last month of life. This study showed a decline in most measured indicators between 2009 and 2013; however, this decline was only statistically significant for the percentage of patients with ICU admission at least once in the last 30 days of life (*P* < .001) and the number of ED visits during last month of life (*P* < .001). This finding is in accordance with many publications that failed to show a significant decline in the percentage of patients receiving a new chemotherapy regimen in the last month of life or who received any chemotherapy within the last 14 days of life.^12,22-25^

Our study also showed that the mean composite score for the aggressiveness of EOL care decreased from 1.33 in 2009 to 1.02 in 2013 (*P* < .01). This finding is in contrast to data reported by Ho et al^5^ from Canada who found an increase in the composite score of aggressiveness of care between 1993 and 2004. Cooke et al^7^ also reported an increasing number of ICU admissions among patients with advanced lung cancer in SEER data from the United States between 1992 and 2005.

Aggressiveness of care at EOL has its known negative effect on patient quality of life as well as its detrimental effect on patient families subjected to a significantly higher rate of psychologic distress compared with those whose relatives or loved ones are not subjected to aggressive management during the last month of life.^1,6^ The decline in aggressive care at EOL in the current study is mostly a result of the opening of the palliative care unit at the end of 2008, the implementation of a do not attempt resuscitation (DNAR) policy in 2011, and the addition of a modified National Comprehensive Cancer Network screening tool for hospitalized patients.

The DNAR policy of our hospital states that DNAR is a clinical decision made by the primary physician and two other oncology physicians. It is not presented to patients or families as a choice; rather, they are informed of the decision. This of course has limited unnecessary intubation and ICU admission for patients with advanced cancer at EOL. The modified NCCCR palliative care referral scoring tool consists of six items, and patients who score a 5 must at least have a palliative care consultation, whereas patients scoring ≥ 7 should be transferred to palliative care. These measures have led to an earlier referral of patients to palliative care beginning in 2012.

In conclusion, the current study shows a decline in the percentage of aggressive care administered to adult patients with cancer in the last month of life from 2009 to 2013. Despite this observed decline, the incidence of aggressive care at EOL in our institution remains significantly higher than that reported in Europe or North America.

Early referral to palliative care, more accurate prognostication near EOL, and availability of chronic care facilities to which patients can be referred should further decrease the percentage of patients who receive aggressive care at our facility.
